# How to model the impact of vaccines for policymaking when the characteristics are uncertain: A case study in Thailand prior to the vaccine rollout during the COVID-19 pandemic

**DOI:** 10.1016/j.vaccine.2023.06.055

**Published:** 2023-07-25

**Authors:** Nantasit Luangasanatip, Chris Painter, Wirichada Pan-ngum, Sompob Saralamba, Tanaphum Wichaita, Lisa White, Ricardo Aguas, Hannah Clapham, Yi Wang, Wanrudee Isaranuwatchai, Yot Teerawattananon

**Affiliations:** a Mahidol Oxford Tropical Medicine Research Unit, Mahidol University, Thailand; b Health Intervention and Technology Assessment Program, Ministry of Public Health, Thailand; c Nuffield Department of Medicine, University of Oxford, United Kingdom; d Saw Swee Hock School of Public Health, National University of Singapore, Singapore

**Keywords:** COVID-19, Vaccine efficacy, COVID-19 vaccines, Mathematical model, Health policy

## Abstract

Thailand faced a dilemma of which groups to prioritise with a limited first tranche of COVID-19 vaccinations in early 2021, at a time when there was low incidence and low mortality in the country. A mathematical modelling analysis was performed to compare the potential short-term impact of allocating the available doses to either the high severity group (over 65-year-olds) or the high transmission group (aged 20–39). At the time of the analysis, there was uncertainty about the precise characteristics of the vaccines available, in terms of their potential impact on transmission and reductions to the severity of infection. As such, a range of vaccine characteristic scenarios, with differing levels of severity and transmission reductions were explored. Using the evidence available at the time regarding severity reduction of infection due to the vaccines, the model suggested that vaccinating high severity group should be the priority if reductions in deaths is the priority. Vaccinating this group was found to have a direct impact on reducing the number of deaths, while the incidence and hospitalisations remained unchanged. However, the model found that vaccinating the high transmission group with a vaccine with sufficiently high protection against infection (more than 70%) could provide enough herd effects to delay the expected epidemic peak, resulting in both case and death reductions in both target groups. The model explored a 12-month time horizon. These analyses helped to inform the vaccination strategy in Thailand throughout 2021 and can inform future modelling studies for policymaking when the characteristics of vaccines are uncertain.

## Highlights

• Mathematical models were used to inform the COVID-19 vaccination strategy in Thailand.
• The model showed the impact of prioritising high severity or high transmission groups.
• The characteristics of the COVID-19 vaccines were uncertain at the time.
• Novel methods were used to understand the impact of this uncertainty.
• The methods may be useful for future vaccination modelling exercises.

## 1 Introduction

At the end of 2020, the first COVID-19 vaccines became available, however questions remained regarding the long-term impact of the available vaccines, and their influence against new variants [1], [2]. Initially the supply of COVID-19 vaccines was extremely tightly controlled, and it seemed unlikely that most countries would have sufficient supplies of vaccines for their entire eligible population in 2021 or even 2022 [3], [4], [5]. In Thailand and in many other countries worldwide, evidence was required to support policy makers decisions regarding how best to allocate limited resources, for example which groups should be prioritised for vaccination and which vaccines should be selected.

Although Thailand was the second country to identify COVID-19 within their population [6], the implementation of NPIs in the first half of 2020 successfully suppressed local transmission throughout the latter part of the year. The tourism sector forms a substantial part of the Thai economy, which suffered greatly as a result of COVID-19. Therefore, the potential value of a COVID-19 vaccine in Thailand differed from many western countries where vaccines were primarily being deployed to reduce morbidity and mortality. However, at the time there was a very high degree of uncertainty around the available vaccines’ transmission blocking effect [7].

The context for COVID-19 vaccination in Thailand in early 2021 was quite specific. The number of available doses for the 12-month period starting from March 2021 was projected to be limited to approximately 31 million people. A very small proportion of the population had been exposed to COVID-19 and, at the end of February 2021, Thailand had 25,951 cases and 83 deaths cumulatively.

The WHO defined COVID-19 vaccine efficacy to include 3 parts: 1) reducing susceptibility; 2) reducing transmission from infected individuals (i.e., reduced infectivity); and 3) reducing severity [8]. As certain characteristics of the vaccine were uncertain, these analyses aimed to explore plausible scenarios of different vaccine characteristics, including efficacy and protection duration. We focussed on comparing vaccines which reduced susceptibility versus vaccines which reduced severity. The target population that it is most beneficial to vaccinate may vary depending on the type of efficacy that a vaccine has. The two subpopulations considered for vaccination in these analyses were the high severity population (the elderly over 65 years old), and the high transmission group (20–39 years old) who were most responsible for transmission of COVID-19 in Thailand. Thailand has a relatively small high severity group with only 13% of the population above the age of 65. The high transmission group (ages 20–39), which is twice as large, is estimated to have approximately double the number of contacts per person [9].

This study shares a modelling experience in the Thai context with the aim of identifying the optimal vaccine strategy to inform policy decisions when the characteristics of vaccination were uncertain. We aimed to identify the optimal COVID-19 vaccination strategy given a set of vaccine characteristics, by exploring the impact that different types of COVID-19 vaccine efficacy can have on outcomes (such as number of new cases and deaths and on different target populations), and comparing trade-offs, and identified thresholds where the optimal vaccination strategy changes. This analysis may be applicable to other countries with similar contexts regarding COVID-19, or future public health emergencies where vaccine modelling may be used to inform consequential public health decisions under uncertainty surrounding the vaccination characteristics.

## 2 Methods

### 2.1 Modelling structure

In this study, we adapted an open source compartmental age-structured model which is based on the SEIR (Susceptible-Exposed-Infective-Recovered) structure developed by the COVID-19 International Modelling Consortium (CoMo consortium) [10], [11], [12]. In the model, the infected compartments were stratified by symptoms, severity, and treatment seeking and access. There are four sub-compartments accounting for different severities of COVID-19 infection which are i) asymptomatic, ii) mild to moderate symptomatic, iii) ICU and iv) ICU with ventilator (see Supplementary A). The CoMo model has also been explained in more detail by Espinosa et al. 2023 [13].

### 2.2 Model parameters and outputs

The model used publicly available country-specific data to define the population structure and mixing contact patterns as model inputs [14]. The model was fitted to the daily new confirmed cases and cumulative deaths reported between 1 Jan–10 May 2020, defined as the first wave of the pandemic in Thailand (see Supplementary B). A 12-month time horizon was used for all model simulations. The primary model outputs were the number of COVID-19 cases by severity, as well as deaths. COVID-19 cases in this case are defined as infections that have tested positive and are reported to public health authorities, as this is the type of case data that the model was fitted to.

### 2.3 Non-pharmaceutical interventions (NPIs)

The modelled effectiveness of NPIs was based on data from their application in Thailand during the first wave, hand hygiene and masking, social distancing, work from home, school closure and travel ban (or border closure) were tracked and incorporated in the model [15], [16], [17]. Further details on this aspect of the model are available in Supplementary B.

### 2.4 Vaccine characteristics and implementation

The introduction of COVID-19 vaccination was expanded from the original CoMo model version 15.0, where we defined the vaccine efficacy by 3 characteristics; i) reduced severity (Efficacy 1), ii) reduced susceptibility (Efficacy 2), iii) Reduced transmission (Efficacy 3). Reduced severity (Efficacy) means reduced symptoms and progression to hospitalisation among the vaccinated. Reduced susceptibility (Efficacy 2) means a direct reduction in infection rate among the susceptible vaccinated group. Reduced transmission (Efficacy 3) means a reduction in force of infection contributed by the vaccinated and infected individuals. Reduced susceptibility and reduced transmission are essentially modelled identically and therefore only Efficacy 1 and Efficacy 2 were explored in the model. It was assumed that only one vaccine was used at a time, and for simplicity partial vaccination (receiving one dose of a two-dose vaccination schedule) was not modelled, or the vaccine can simply be thought of as a single dose schedule. The coverage of vaccine was modelled based on the government’s initial vaccine target coverage of 70%, to be reached by the end of 2021 [18]. The linear increment of monthly coverage was assumed allowing reasonable comparison between vaccination options. It was also assumed that vaccine effectiveness was equal across all population groups and did not consider vaccine side-effects. As the protective duration of all the vaccines available is still unknown, we assumed two scenarios, a short-term and long-term duration of protection of 6 months or 1 year, respectively.

### 2.5 Base case and scenario analyses

There were two base case scenarios set to explore further vaccination strategies based on the uncertainty around the situation of COVID-19 pandemic. Two degrees of NPI levels were assumed in the model (Table 1), and they were applied throughout the entire model time horizon. The model time horizon of vaccination impact from 1st March 2021 to 1st March 2022. In both scenarios, vaccine efficacies of 70% and 90% and protection durations of 6 months and 1 year were explored.

**Table 1:** Base case non-pharmaceutical intervention scenario descriptions.

	Base case scenarios	
	Scenario A	Scenario B
Intervention	Adherence	Adherence
Hand washing	15%	15%
Mask wearing	50%	50%
Social distancing	30%	90%

The analyses aimed to address the following questions: Issue 1): What is the impact of different types of vaccine efficacy on which subpopulations should be prioritised for COVID-19 vaccination? Issue 2) Is vaccine efficacy or protection duration a more preferable vaccine characteristic? Issue 3: What is the impact of combined efficacy types on which subpopulations should be prioritised for COVID-19 vaccination?

Issues 1 and 2 were addressed by assuming that the available vaccine had only one type of efficacy, by either reducing susceptibility to infection, or the severity of the infection. In contrast, the analysis for Issue 3 assumed that the vaccines could have more than one type of vaccine efficacy, with a trade-off in vaccine efficacy between reduced susceptibility and reduced severity at a fixed level of combined efficacy (either 70% or 90%) using the formula: *(1 – overall efficacy) = (1 – eff[1])×(1 – eff[2])*. A separate analysis was performed where the reduction in severity from vaccination was fixed at 70% and the reduction in susceptibility was varied incrementally between 0% and 70%. The outputs of this scenario analysis were the percentage reduction in deaths and cases compared to the baseline, when the vaccine was implemented in different population groups and under different NPI levels. The rationale for fixing the reduction in severity and varying the reduction in susceptibility was that there would be more uncertainty surrounding the reduction in susceptibility due to the design of clinical trials. The model code and the data used in this paper can be found at https://github.com/slphyx/CoVac19TH.

## 3 Results

Issue 1: What is the impact of different types of vaccine efficacy on which subpopulations should be prioritised for COVID-19 vaccination?

**Fig. 1:**
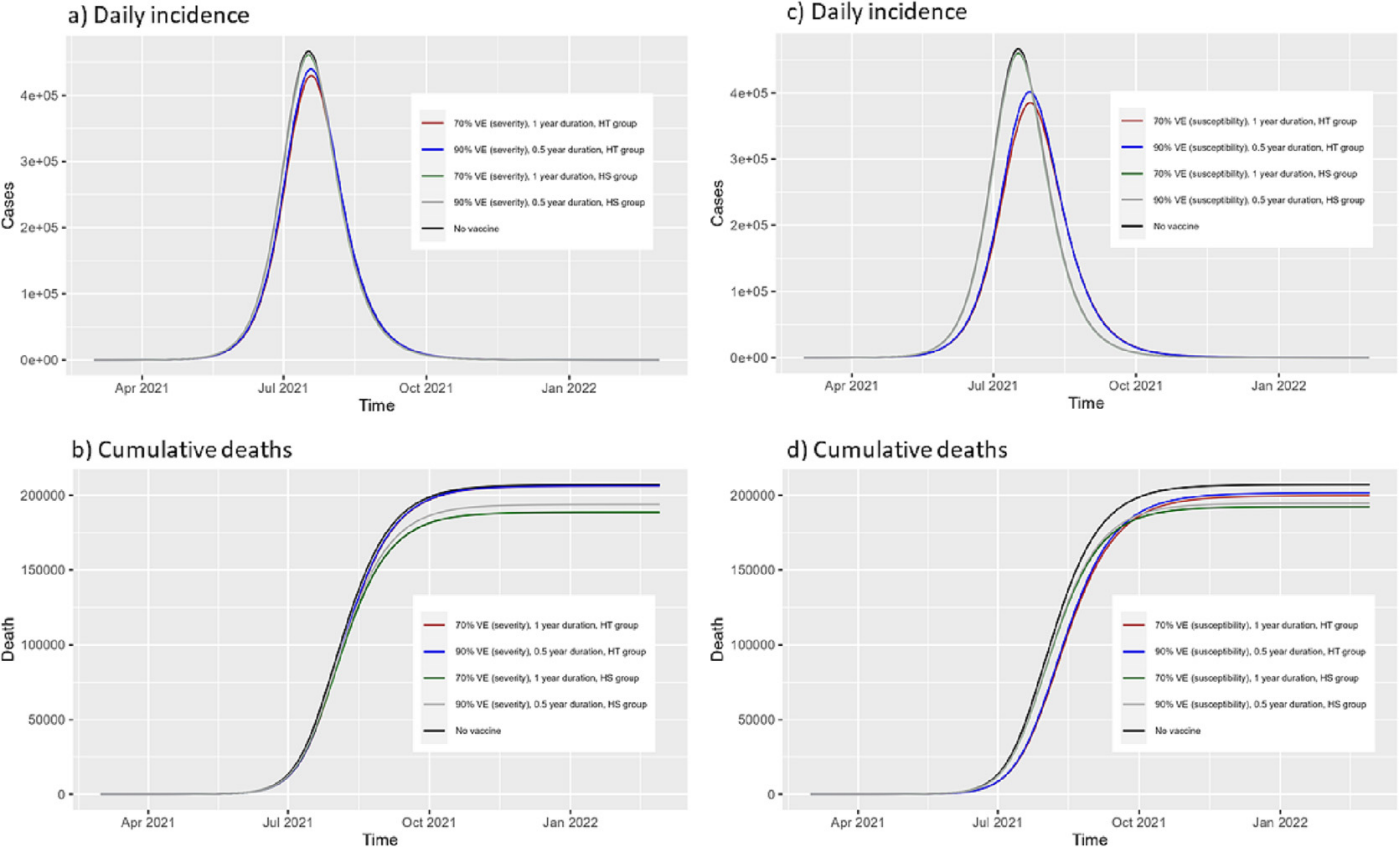
Scenario A (less strict social distancing measures) – Comparison vaccines that reduce severity (a-b) and reduce susceptibility (c-d), 70% efficacy with 1 year duration vs 90% efficacy with 0.5 year in both high transmission and high severity groups, Abbreviations: HS, high severity; HT, high transmission.

**Fig. 2:**
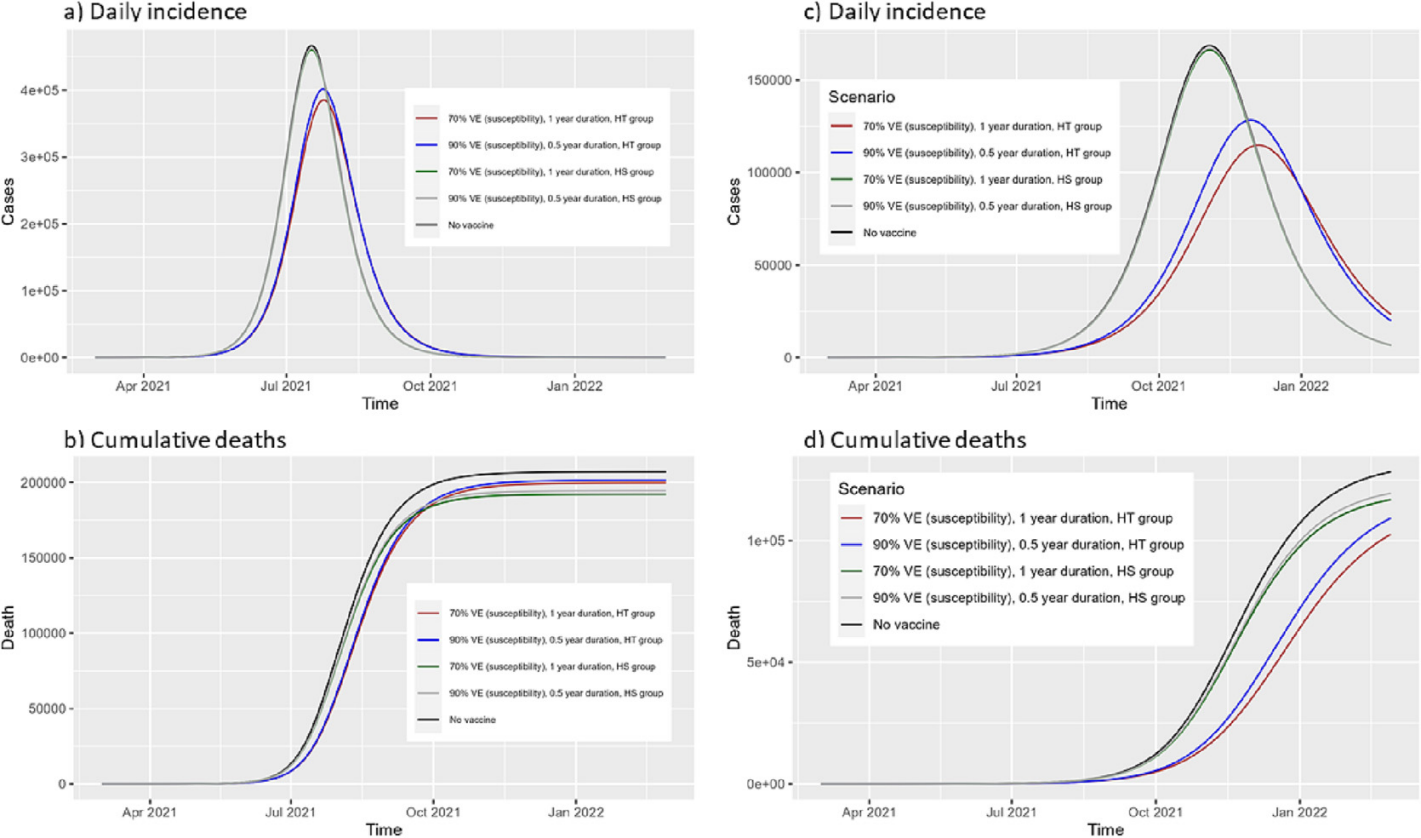
Scenario B (more strict social distancing measures) – Comparison between vaccines that reduce severity (a-b) and reduce susceptibility (c-d), 70% efficacy with 1 year duration vs 90% efficacy with 0.5 year in both high transmission and high severity groups, Abbreviations: HS, high severity; HT, high transmission.

The results of the base case analyses for scenarios A and B are displayed in Fig. 1 and Fig. 2, respectively. The results displayed in Fig. 1 show that in Scenario A, where social distancing measures were more relaxed, vaccinating the high severity group was projected to result in fewer deaths than vaccinating the high transmission group (though the incidence was higher) for both severity and susceptibility reducing vaccines. Fig. 1 and Fig. 2 a) and b) show results when the efficacy of the vaccine reduces severity, resulting in a proportional reduction in symptoms and hospitalisation in infected and vaccinated people. Whereas, c) and d) show results when the efficacy of the vaccine reduces susceptibility, resulting in a direct reduction in transmissibility, reducing the new infections among vaccinated susceptible individuals. Conversely, Fig. 2 shows that when social distancing measures were more stringent, that the optimal group to vaccinate differed depending on the type of vaccine efficacy in action. If the available vaccine reduced severity only, then vaccinating high severity individuals resulted in fewer deaths. However, if the vaccine reduced susceptibility then vaccinating the high transmission group resulted in fewer deaths.

Issue 2: Is vaccine efficacy or protection duration a more preferable vaccine characteristic?

When comparing between the lower efficacy but longer duration of protection (70% and 1 year) to the higher efficacy but shorter duration of protection (90% and 6 months), the results were consistent across all scenarios and efficacy types. The longer duration of protection and lower efficacy vaccines prevented a greater or comparable number of cases and deaths than vaccines that are 90% effective for 6 months. When vaccines are targeted to elderly adults, trading off efficacy for duration of protection did not affect the number of cases. When social distancing is less stringent and vaccines are targeted to young adults, trading off efficacy for duration of protection did not affect the number of deaths.

Issue 3: What is the impact of combined efficacy types on which subpopulations should be prioritised for COVID-19 vaccination?

The results from Analysis 1 showed that in a situation where a vaccine only had a single type of efficacy, the decision on who should be prioritised could vary depending on the type of vaccine efficacy and strength of social distancing measures in place.

**Fig. 3:**
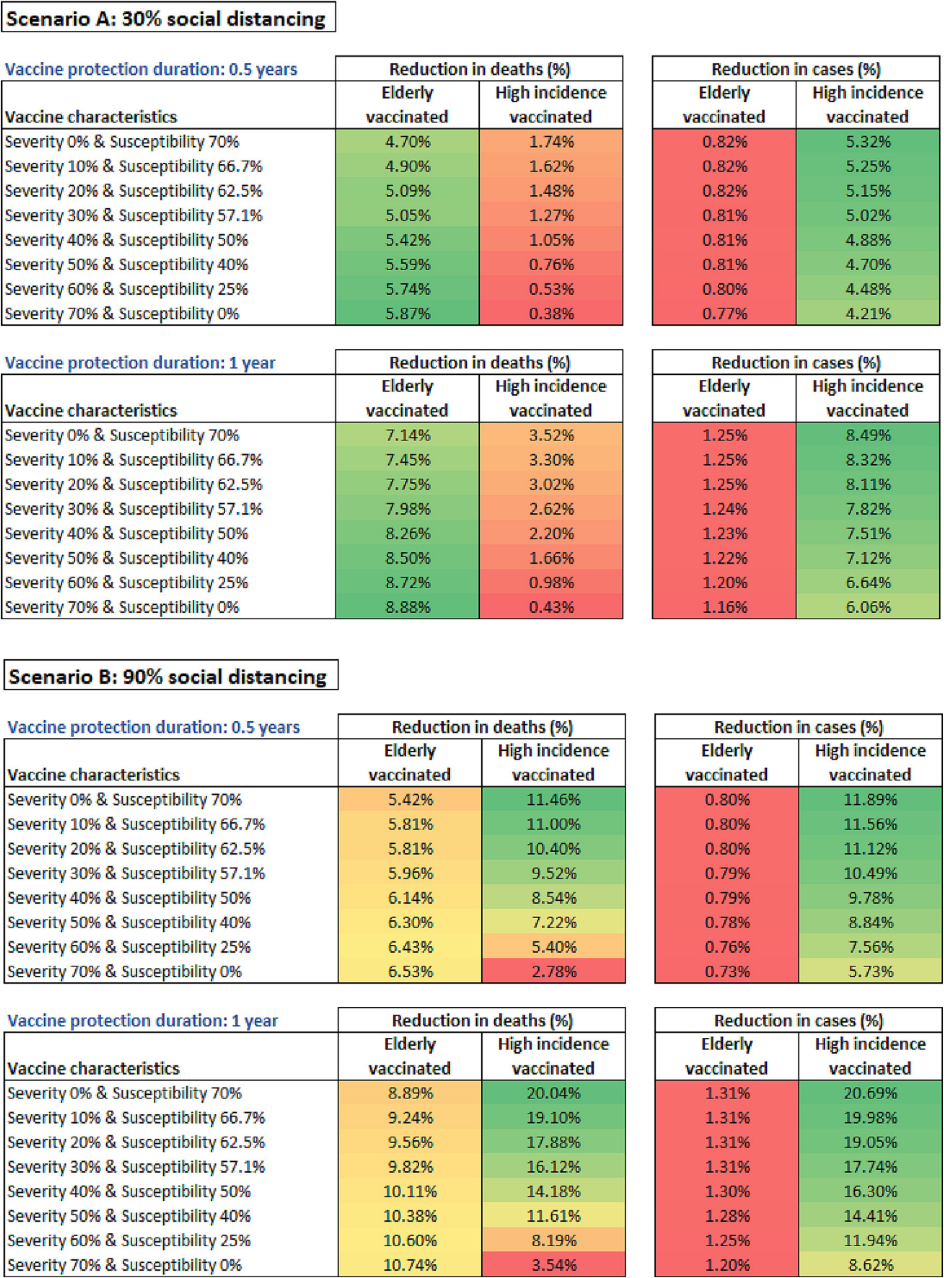
Combined vaccine efficacy of 70% with variations in reductions in severity and susceptibility.

To further interrogate this question, we considered scenarios where the vaccine had more than one type of efficacy. In these analyses, different combinations of reduced susceptibility and reduced severity levels as a result of vaccination were explored, as displayed in Fig. 3. In scenarios where social distancing was reduced by 30%, if the policy aim was the minimisation of deaths, then the results showed that the preferred vaccine strategy should be to prioritise the high severity group irrespective of the combination of vaccine efficacies. Conversely, if the aim was to reduce cases then the high transmission group should be prioritised. Whereas in the scenario where social distancing was reduced by 90%, the results showed that vaccinating the high transmission group could be preferred if the policy aim was minimising deaths in certain scenarios. Vaccinating the high transmission group was more preferable to vaccinating the elderly in scenarios where the vaccine caused greater reductions in susceptibility and had a lesser impact on severity. In all scenarios, if case reduction was the policy aim, vaccinating the high transmission group was preferable. The conclusions were consistent regardless of the protection duration of the vaccine.

**Fig. 4:**
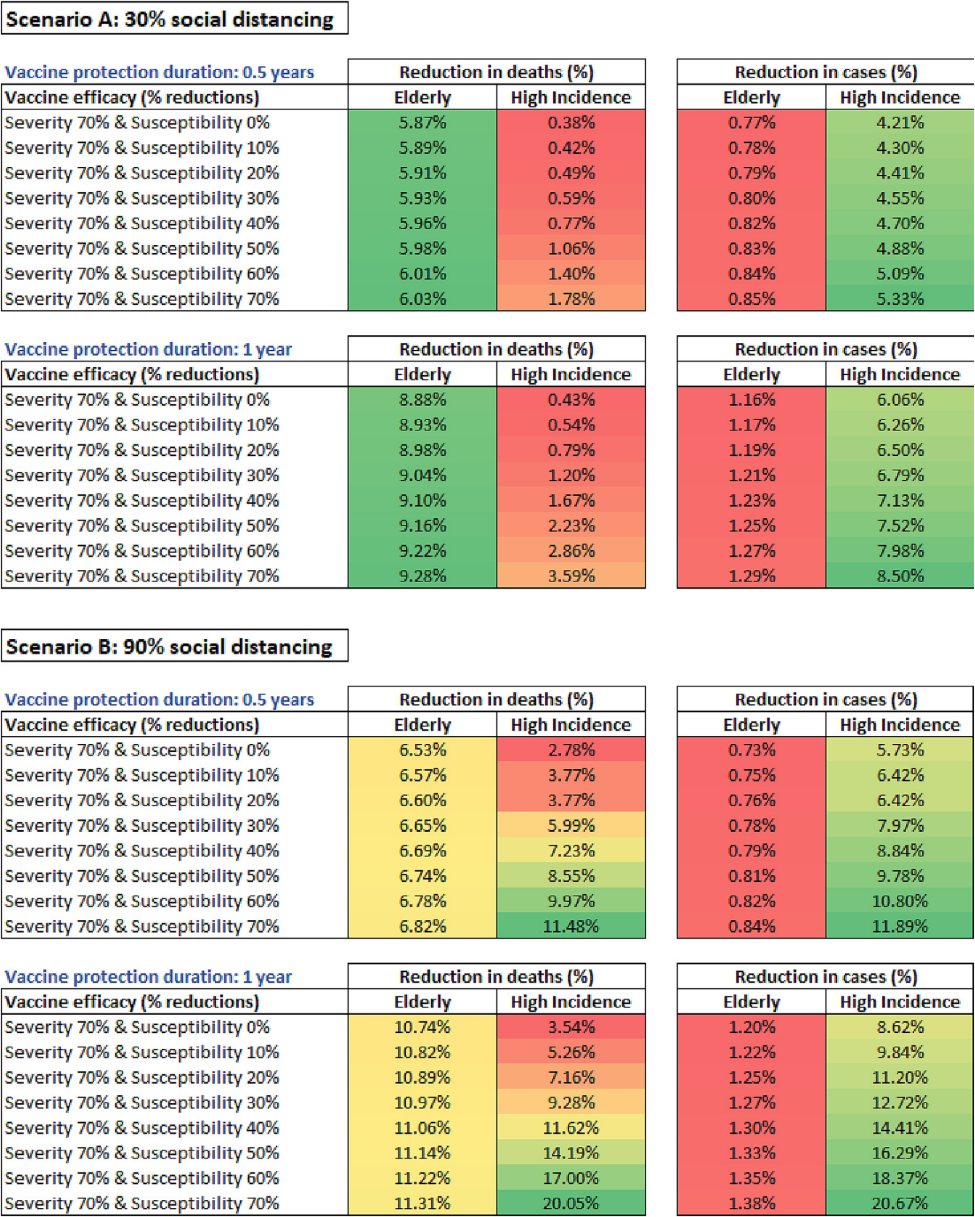
Reduction in severity vaccine efficacy fixed at 70% with reductions in susceptibility varied between 0% and 70%.

Fig. 4 shows the results of the scenario analyses where reductions in severity from vaccination were fixed at 70%, and reductions in susceptibility were varied incrementally between 0% and 70%. The results show that when levels of social distancing were reduced by only 30%, the conclusion is consistent that if death minimisation was the policy aim then the elderly group should be prioritised for vaccination. Whereas when case reduction was the aim then the high transmission group should be prioritised. However, when social distancing was reduced by 90%, when the reduction in susceptibility was 40% or greater, vaccinating the high transmission group resulted in fewer deaths in both the 0.5 and 1 year protection duration scenarios. When the reduction in susceptibility was below 40%, vaccinating the elderly resulted in fewest deaths. In all scenarios vaccinating the high transmission group was the optimal policy for reducing the number of cases.

## 4 Discussion

This study demonstrates how researchers and mathematical modellers can aim to inform important policymaking decisions for vaccine allocation programs when there is a large degree of uncertainty about the effects of vaccines, particularly by considering characteristics of vaccination which at the time were unknown, and in a setting where there was low COVID-19 incidence and mortality. The analyses performed aided decision makers by providing the details, benefits, risks and expected outcomes associated with each policy option. Given the difficulty of forecasting absolute projections for COVID-19 beyond the short-term, the modelling exercise focussed on the comparative impacts between different policy interventions and extensive scenario analyses.

The results from Analysis 1 shows that reductions in susceptibility or transmission as a result of vaccination is an important consideration for a country like Thailand, which had limited or low local transmission of COVID-19 at the time. As evidence continued to emerge and new information became available regarding the vaccine’s impact on susceptibility and transmission, the results from this paper allowed policymakers to quickly re-evaluate vaccine choices. Moreover, the type of vaccine efficacy was not the only important characteristic to distinguish between the available vaccines; vaccine protection efficacy and duration were also important characteristics in order to maximise the population health impact from vaccination.

The WHO SAGE’s prioritisation roadmap of COVID-19 vaccines would recommend that in the Thai context the high severity group should be prioritised for vaccination, as a country with localised or limited transmission [19]. However, this study interrogated that advice by considering the potential benefits of vaccinating alternative priority groups under various scenarios of characteristics of COVID-19 vaccine efficacy and NPI interventions. The high severity group remained the priority when aiming to minimise deaths. However, this study showed that when the strength of NPIs in place are high, and the thus incidence of COVID-19 remained low, then vaccinating the high transmission population could be preferable as this group played a larger role in transmission in Thailand at that time. At the time there was emerging evidence that vaccines may be able to reduce transmission (reduce infectivity) by up to 67% [20], although the confidence interval for this estimate included 0% so strategies depending on this value should be considered with caution.

Thailand’s target vaccine supply was expected to be enough to vaccinate 50% of the population in the first year of their availability [21]. Due to this expectation of limited supply, prioritisation of groups for vaccination was an unavoidable decision for policymakers. The results of this study were used for policy discussions in Thailand regarding the prioritisation of groups for a COVID-19 vaccine amongst the general population. Health professionals had already been prioritised for vaccination, based on ethical justifications and in line with the WHO SAGE’s prioritisation roadmap of COVID-19 vaccines [19]. Although these decisions are ultimately left to policymakers, it is important for researchers to produce evidence as presented in this study to inform policy debates. The methods demonstrated here can be used to inform future mathematical modelling exercises for policymaking to optimise the use of vaccines when their characteristics are uncertain.

As the mathematical model was highly specific to Thailand, the findings on the impact of COVID-19 vaccination are most applicable to Thailand in early 2021 or similar settings with well-controlled community transmission. As this study shows, it is important for countries with different demographics and outbreak profiles to conduct their own studies to contextualise evidence for informing policy. This is reinforced by a study that was conducted at a similar time, by Moore et al., which found that prioritising the vaccination of high severity individuals was the most effective strategy for reducing the number of deaths in the UK, where there was active local transmission, a proportionally larger high severity population group, and higher case-fatality rates [22].

The vaccines considered in this study were all hypothetical, so the results of this study could not be used to advocate for individual vaccines. A benefit of evaluating vaccines in this way is that it allowed consideration of a broader set of vaccine characteristics than was available for the vaccines that were obtainable at the time. Ultimately, Thailand decided to initially prioritise the high severity group for vaccination. This approach also facilitated the evaluation of new vaccines, or new information on existing vaccines, as these became available. Furthermore, many countries were unable to rely solely on supplies of a single vaccine, and therefore evaluations that considered only one vaccine were not suitable for policymakers.

While the effectiveness of NPIs could not be reliably quantified [23], as these were implemented during a period where the number of reported cases and deaths in the early phase of the pandemic were extremely limited in Thailand [24], sensitivity analyses were performed around the parameter to demonstrate the robustness of the model outputs of Fig. 1, shown in Fig. S2. Incidence was much more robust in comparison to mortality. However, the conclusion on the subpopulation for vaccine prioritisation was consistent despite the uncertainties surrounding NPI effectiveness.

## Declaration of Competing Interest

The authors declare that they have no known competing financial interests or personal relationships that could have appeared to influence the work reported in this paper.

## Data Availability

The model code and the data used in this paper can be found at https://github.com/slphyx/CoVac19TH. Additional details are also available in the supplementary materials.
