# Supplementary material for: How to model the impact of vaccines for policymaking when the characteristics are uncertain: A case study in Thailand prior to the vaccine rollout during the COVID-19 pandemic
Source: Vaccine. 2023 Jul 25;41(33):4854–60. doi: 10.1016/j.vaccine.2023.06.055 (PMC10281228; doi:10.1016/j.vaccine.2023.06.055)
Supplement: Supplementary data 1 [file mmc1.docx]

**Supplementary Materials**

1. **Transmission dynamic model**
2. **Model fitting and parameter estimation**

# **Supplementary A– Transmission dynamic model**

**Figure A1: Model structure**

**Figure A2: Observed reported cases with the tracked timeline of non-pharmaceutical interventions implemented during the first outbreak in Thailand**

1.
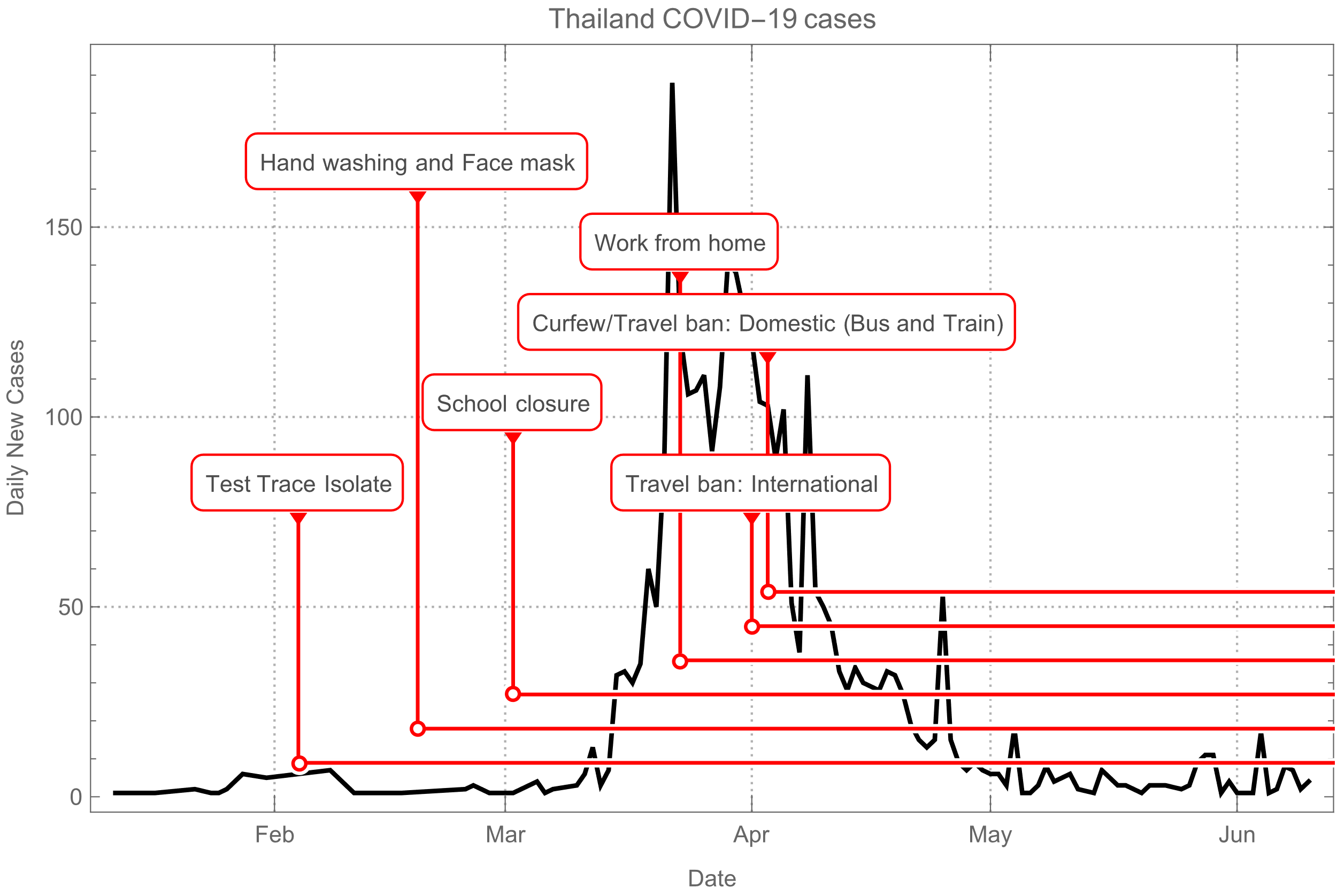


# **Supplementary B – Model fitting and parameter estimations**

**Table B1: List of the parameters with values and references**

| **Population Parameters** | **Value** | **Unit** | **References** |
| --- | --- | --- | --- |
| Initial population | 69,799,978 |  |  |
| **Virus Parameters** | **Value** | **Unit** | **References** |
| Probability of infection given a contact | 0.029 |  | from fitting the model to the 1^st^ incidence data |
| Relative infectiousness of incubation phase: | 10 | % | (Jing, Liu et al. 2020) |
| Average incubation period: | 3.5 | days | (Bi, Wu et al. 2020, European centre for disease prevention and control 2020, Khalili, Karamouzian et al. 2020, Linton, Kobayashi et al. 2020) |
| Average duration of symptomatic infection period: | 4.5 | days | (Linton, Kobayashi et al. 2020) |
| Average duration of immunity: | 1 | year |  |
| Probability upon infection of developing clinical symptoms: | 50 | % | % (Yanes-Lane, Winters et al. 2020, Alene, Yismaw et al. 2021, Oran and Topol 2021) |
| Probability upon hospitalisation of requiring ICU admission: | 25 | % | (Chang, Elhusseiny et al. 2021) |
| Probability upon admission to the ICU of requiring a ventilator: | 69 | % | (Chang, Elhusseiny et al. 2021) |
| Reporting of all aymptomatic infections | 2.5 | % |  |
| Reporting of all symptomatic infections | 90 | % |  |
| Reporting of all infections requiring hospitalisation | 95 | % |  |
| **Hospitalisation parameters** | **Value** | **Unit** | **References** |
| Relative percentage of regular daily contacts when hospitalised | 5 | % | Assumption |
| Probability of dying when hospitalised: | 40 | % | (Petrilli, Jones et al. 2020) |
| Probability of dying when denied hospitalisation: | 60 | % | (Petrilli, Jones et al. 2020) |
| Probability of dying when admitted to ICU: | 50 | % | (Lewnard, Liu et al. 2020, Petrilli, Jones et al. 2020) |
| Probability of dying when admission to ICU denied: | 50 | % | (Petrilli, Jones et al. 2020) |
| Probability of dying when ventilated: | 70 | % | (Petrilli, Jones et al. 2020) |
| Probability of dying when ventilator denied: | 70 | % | (Petrilli, Jones et al. 2020) |
| Duration of hospitalised infection: | 12 | days | (National Health Security Office 2020) |
| Duration of denied hospitalisation infection: | 12 | days | (National Health Security Office 2020) |
| Duration of ICU infection: | 20 | days | (National Health Security Office 2020) |
| Duration of denied ICU infection: | 20 | days | (National Health Security Office 2020) |
| Duration of ventilated infection: | 20 | days | (National Health Security Office 2020) |
| Duration of denied ventilation infection: | 20 | days | (National Health Security Office 2020) |

| **NPIs** | **Date Start** | **Date End** | **Coverage (%)** | **Efficacy**  **(%)** |
| --- | --- | --- | --- | --- |
| Hand hygiene & mask wearing | 15/2/2020 | 20/3/2020 | 50 | 20 |
| Hand hygiene & mask wearing | 21/3/2020 | 30/6/2020 | 80 | 20 |
| School Closures | 1/3/2020 | 30/6/2020 | 90 | 100 (Home contacts inflation due to school closure 15%) |
| Social Distancing | 1/1/2020 | 27/3/2020 | 15 | 100 |
| Social Distancing | 28/3/2020 | 30/6/2020 | 95 | 100 |
| International Travel Ban | 21/3/2020 | 5/4/2020 | 40 | 100 |
| International Travel Ban | 6/4/2020 | 30/6/2020 | 90 | 100 |
| Working at Home | 21/3/2020 | 16/5/2020 | 70 | 85 (Home contacts inflation due to working from home 10%) |
| Working at Home | 17/5/2020 | 30/6/2020 | 50 | 85 (Home contacts inflation due to working from home 10%) |
| Mass Testing | 21/3/2020 | 1/9/2020 | 5 | 85% (sensitivity) |
| Self-isolation if Symptomatic | 20/3/2020 | 2/6/2020 | 50 | 80% (test sensitivity) |

**Table B2: Age-specific hospitalization and fatality rate**

| Age_category | Age-based relative fatality rate (%) | Age-stratum-specific hospitalization (proportion of all (asymptomatic + symptomatic) infections that lead to hospitalisation) (%) |
| --- | --- | --- |
| 0-5 y.o. | 0.57 | 0.52 |
| 5-10 y.o. | 0.57 | 0.52 |
| 10-15 y.o. | 0.57 | 0.52 |
| 15-20 y.o. | 0.57 | 0.52 |
| 20-25 y.o. | 2.85 | 0.95 |
| 25-30 y.o. | 2.85 | 0.95 |
| 30-35 y.o. | 6.28 | 1.65 |
| 35-40 y.o. | 6.28 | 1.65 |
| 40-45 y.o. | 7.99 | 2.86 |
| 45-50 y.o. | 7.99 | 2.86 |
| 50-55 y.o. | 16.54 | 5.63 |
| 55-60 y.o. | 16.54 | 5.63 |
| 60-65 y.o. | 34.71 | 10.92 |
| 65-70 y.o. | 34.71 | 10.92 |
| 70-75 y.o. | 35.50 | 18.20 |
| 75-80 y.o. | 35.50 | 18.20 |
| 80-85 y.o. | 100.00 | 27.39 |
| 85-90 y.o. | 100.00 | 27.39 |
| 90-95 y.o. | 100.00 | 27.39 |
| 95-100 y.o. | 100.00 | 27.39 |
| 100+ y.o. | 100.00 | 27.39 |

**Table B3: Demographic population, birth and death rate for Thailand (Reference: UN2019 Revision of World Population Prospects https://population.un.org/wpp/)**

| Age_category | Population | Number of births per person (ie 0.5* births per woman) per day | Deaths per person per day |
| --- | --- | --- | --- |
| 0-4 y.o. | 3,596,052 | 0 | 5.0051E-06 |
| 5-9 y.o. | 3,843,780 | 0 | 7.5203E-07 |
| 10-14 y.o. | 4,113,805 | 0 | 1.1683E-06 |
| 15-19 y.o. | 4,378,506 | 6.30376E-05 | 3.1E-06 |
| 20-24 y.o. | 4,807,904 | 0.0001026 | 2.8347E-06 |
| 25-29 y.o. | 4,822,404 | 0.000105626 | 2.9941E-06 |
| 30-34 y.o. | 4,466,694 | 9.60249E-05 | 4.5864E-06 |
| 35-39 y.o. | 4,763,033 | 4.42185E-05 | 7.165E-06 |
| 40-44 y.o. | 5,308,840 | 1.07929E-05 | 9.5791E-06 |
| 45-49 y.o. | 5,605,417 | 1.48326E-06 | 1.221E-05 |
| 50-54 y.o. | 5,598,953 | 0 | 1.5486E-05 |
| 55-59 y.o. | 5,082,441 | 0 | 2.0401E-05 |
| 60-64 y.o. | 4,367,653 | 0 | 2.819E-05 |
| 65-69 y.o. | 3,256,703 | 0 | 3.914E-05 |
| 70-74 y.o. | 2,282,338 | 0 | 6.3727E-05 |
| 75-79 y.o. | 1,584,230 | 0 | 0.00010856 |
| 80-84 y.o. | 1,070,912 | 0 | 0.00016786 |
| 85-89 y.o. | 563,683 | 0 | 0.00026194 |
| 90-94 y.o. | 225,198 | 0 | 0.00036263 |
| 95-99 y.o. | 52,666 | 0 | 0.00064432 |
| 100+ y.o. | 8,766 | 0 | 0.0038711 |

**Figure B1: Model fitting using incidence and cumulative deaths data from 2020**


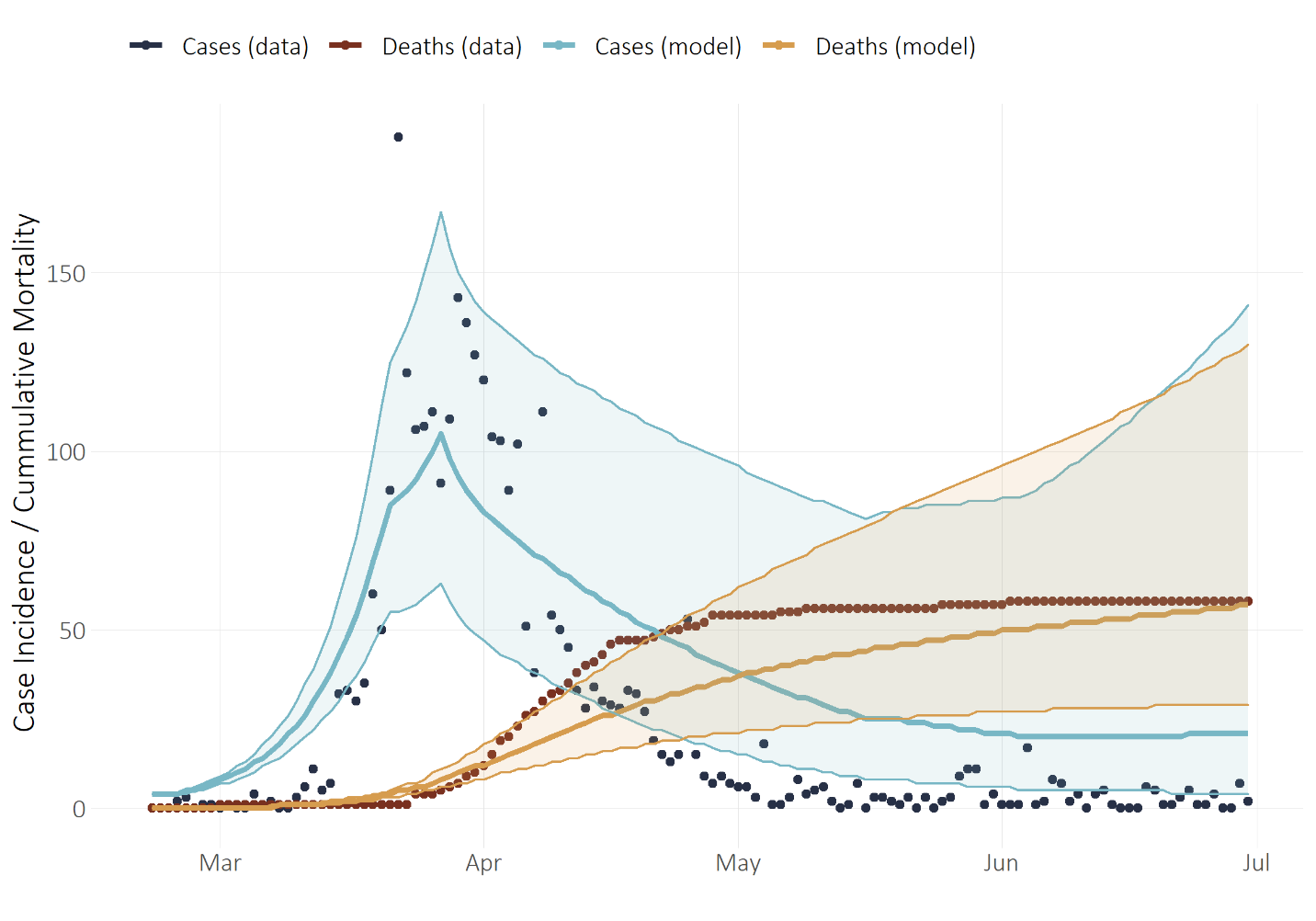


# References

Alene, M., et al. (2021). "Magnitude of asymptomatic COVID-19 cases throughout the course of infection: A systematic review and meta-analysis." **16**(3): e0249090.

Bi, Q., et al. (2020). "Epidemiology and Transmission of COVID-19 in Shenzhen China: Analysis of 391 cases and 1,286 of their close contacts." MedRxiv.

Chang, R., et al. (2021). "COVID-19 ICU and mechanical ventilation patient characteristics and outcomes—A systematic review and meta-analysis." **16**(2): e0246318.

European centre for disease prevention and control (2020). "Coronavirus disease 2019 (COVID-19) pandemic increased transmission in the EU/EEA and the UK seventh update." from <https://www.ecdc.europa.eu/sites/default/files/documents/RRA-seventh-update-Outbreak-of-coronavirus-disease-COVID-19.pdf>.

Jing, Q.-L., et al. (2020). "Household secondary attack rate of COVID-19 and associated determinants in Guangzhou, China: a retrospective cohort study." **20**(10): 1141-1150.

Khalili, M., et al. (2020). "Epidemiological characteristics of COVID-19: a systematic review and meta-analysis." Plos One **148**.

Lewnard, J. A., et al. (2020). "Incidence, clinical outcomes, and transmission dynamics of hospitalized 2019 coronavirus disease among 9,596,321 individuals residing in California and Washington, United States: a prospective cohort study." MedRxiv.

Linton, N. M., et al. (2020). "Incubation period and other epidemiological characteristics of 2019 novel coronavirus infections with right truncation: a statistical analysis of publicly available case data." J Clin Med **9**(2): 538.

National Health Security Office (2020). "E-claim database of in-patient department under the Universal Coverage Scheme."

Oran, D. P. and E. J. J. A. o. i. m. Topol (2021). "The proportion of SARS-CoV-2 infections that are asymptomatic: a systematic review." **174**(5): 655-662.

Petrilli, C. M., et al. (2020). "Factors associated with hospitalization and critical illness among 4,103 patients with COVID-19 disease in New York City." medRxiv.

Yanes-Lane, M., et al. (2020). "Proportion of asymptomatic infection among COVID-19 positive persons and their transmission potential: A systematic review and meta-analysis." **15**(11): e0241536.
